# Research Progress of Biliary Tract Cancers

**DOI:** 10.3390/cancers13040919

**Published:** 2021-02-22

**Authors:** Lionel Aurelien A. Kankeu Fonkoua, Amit Mahipal

**Affiliations:** Division of Medical Oncology, Mayo Clinic, 200 First Street SW, Rochester, MN 55905, USA; KankeuFonkoua.Lionel@mayo.edu

**Keywords:** cholangiocarcinoma, gallbladder cancer, chemotherapy, fibroblast growth factor receptor

This series of nine articles (three original articles, six reviews) is presented by international leaders in biliary tract cancers (BTC). BTCs are a rare but heterogeneous group of malignancies with dismal overall prognosis and comprised of intrahepatic cholangiocarcinoma (CCA), extrahepatic CCA, and gallbladder cancer (GBC). Surgery is the only potentially curative treatment, but the majority (~65%) of patients present at unresectable stage at the time of diagnosis, with a 5-year overall survival (OS) rate of less than 5% [1].

The high rate of unresectability has generated considerable interest in orthotopic liver transplantation (OLT). While OLT is currently not recommended outside of clinical trial for intrahepatic cholangiocarcinoma (iCCA) due to high tumor recurrence rates [2,3,4], it remains a viable option for patients with perihilar cholangiocarcinoma (pCCA) meeting strict eligibility criteria. Excellent outcomes have been achieved in highly selected pCCA patients with the Mayo protocol, using neoadjuvant chemoradiation and a brachytherapy boost followed by liver transplantation [5,6]. Long-term survival after OLT has been shown to be better in patients with pCCA arising in the background of primary sclerosing cholangitis (PSC) when compared to de novo pCCA (74% vs. 58% 5-year OS rate, respectively), and in this thematic issue, Azad et al. review the likely clinical factors contributing to these divergent post-transplant survival outcomes, and offer insights into how further advances may improve patient selection and survival [7].

While the surgical management of T3/T4 GBC has been controversial, primarily due to a high rate of lymph node positivity and occult metastatic disease, successful extensive radical resections can be achieved in selected patients [8]. In this thematic issue, Higuchi et al. explored the poor prognostic factors affecting long-term surgical outcomes in this patient population [9]. After examining 157 cases of resected stage III and IV GBC, they concluded that the presence of two or more of the following preoperative factors confers a poor prognosis: hepatic invasion ≥ 5 mm, invasion of the left margin or the entire area of the hepatoduodenal ligament, or ≥4 lymph node metastases. This suggests that novel strategies expanding beyond surgery alone will be needed to improve outcomes in this patient population. Neoadjuvant treatment can potentially be considered in selected high-risk patients to downstage the tumor and potentially decrease the probability of recurrence.

For most of the patients with BTC that are ineligible for potentially curative therapies, cytotoxic chemotherapy remains the cornerstone of management, despite dismal survival outcomes. To date, the combination of gemcitabine and cisplatin has been established as the preferred first-line regimen [10]. The results of ongoing trials attempting to improve upon this standard of care doublet are eagerly awaited, including the phase III SWOG S1815 study investigating the triplet of gemcitabine, cisplatin and nab-paclitaxel. Other potential first-line options include GEMOX, GEMCAP, FOLFOX, or gemcitabine monotherapy, depending on clinical setting and patient-related factors. In this thematic issue, Markussen et al. present the results of a randomized phase II trial investigating the efficacy of the combination of oxaliplatin, gemcitabine and capecitabine to standard of care gemcitabine plus cisplatin [11]. While the triplet was more convenient in terms of the infusion time and number of visits, gemcitabine plus cisplatin demonstrated superior survival benefits. Data on options beyond first-line have historically been scarce, although recent data from the phase III ABC-06 study support the use of FOLFOX [12]. In this thematic issue, Pape et al. assessed the efficacy of the novel topoisomerase II inhibitor CAP7.1 in patients with advanced BTC, whose disease had progressed on prior chemotherapy [13]. The disease control rate was better compared to best supportive care (BSC), with an associated greater time to disease progression, suggesting that CAP7.1 warrants further investigation in a larger randomized trial.

While an anatomically diverse group, recent molecular profiling efforts of BTCs have revealed a wealth of genomic alterations with prognostic and therapeutic implications. These actionable alterations have been the subject of ongoing clinical investigations, with the most promising targets to date being fibroblast growth factor receptor (FGFR), isocitrate dehydrogenase (IDH) 1 and 2, BRAF, mismatch repair proteins and HER2-neu [14]. Most recently, the FDA approved pemigatinib, a selective potent oral inhibitor of FGFR1- 3, for the treatment of previously treated, unresectable, locally advanced or metastatic CCA with FGFR2 fusion or other rearrangement. Guidelines are being developed for the effective management of FGFR inhibitor-associated toxicities [15]. While pemigatinib is the only FDA-approved targeted therapy to date, this is expected to change soon, as we expand our understanding of the molecular pathways and therapeutic resistance mechanisms involved in BTCs. Several papers in this series provide a critical review of the molecular characterization of BTC, while elaborating on the molecular features that can be translated into therapeutic biomarkers and targets for clinical use [16,17]. Wijetunga and colleagues reported findings of a systematic review aiming to identify biomarkers suitable for theranosis, using a novel bioinformatics approach [18]. They highlight existing validated markers of CCA that can be used for the future development of targeted theragnostic delivery systems.

CCA exhibits a highly desmoplastic stroma that plays a key role in CCA tumorigenesis, invasiveness and therapeutic resistance through remodeling of the tumor extracellular matrix and cross-talk with proinflammatory immune subsets [19,20]. Meanwhile, Malenica et al. discuss recent research progress in the immunological characterization of BTCs and its implications for the development of novel immune-based therapies. Caligiuri et al. review the role of chemokines in the regulation of CCA development and progression, and the modulation of angiogenesis, metastasis and immune control [21,22]. They summarize the most recent findings on the role played by chemokine-induced signals in driving CCA malignancy and discuss the potential role of chemokines and their receptors as possible biomarkers and/or therapeutic targets for hepatobiliary cancers.

It is our hope that this thematic issue provides updated information on the evolving treatment paradigms and research progress in the field of BTCs. This series of unique articles highlights the rapidly growing precision medicine efforts and reflects on future directions which may lead to improved outcomes for patients with this lethal disease.

## References

[1] Siegel R.L., Miller K.D., Jemal A. (2017). Cancer Statistics, 2017. CA Cancer J. Clin..

[2] Ghali P., Marotta P.J., Yoshida E.M., Bain V.G., Marleau D., Peltekian K., Metrakos P., Deschenes M. (2005). Liver transplantation for incidental cholangiocarcinoma: Analysis of the Canadian experience. Liver Transpl..

[3] Becker N.S., Rodriguez J.A., Barshes N.R., O’Mahony C.A., Goss J.A., Aloia T.A. (2008). Outcomes analysis for 280 patients with cholangiocarcinoma treated with liver transplantation over an 18-year period. J. Gastrointest. Surg..

[4] Lunsford K.E., Javle M., Gaber A.O., Vauthey J.N., Ghobrial R.M. (2018). Liver transplantation for locally advanced intrahepatic cholangiocarcinoma—Authors’ reply. Lancet Gastroenterol. Hepatol..

[5] Darwish Murad S., Kim W.R., Harnois D.M., Douglas D.D., Burton J., Kulik L.M., Botha J.F., Mezrich J.D., Chapman W.C., Schwartz J.J. (2012). Efficacy of neoadjuvant chemoradiation, followed by liver transplantation, for perihilar cholangiocarcinoma at 12 US centers. Gastroenterology.

[6] Rea D.J., Heimbach J.K., Rosen C.B., Haddock M.G., Alberts S.R., Kremers W.K., Gores G.J., Nagorney D.M. (2005). Liver transplantation with neoadjuvant chemoradiation is more effective than resection for hilar cholangiocarcinoma. Ann. Surg..

[7] Azad A.I., Rosen C.B., Taner T., Heimbach J.K., Gores G.J. (2020). Selected Patients with Unresectable Perihilar Cholangiocarcinoma (pCCA) Derive Long-Term Benefit from Liver Transplantation. Cancers.

[8] Onoyama H., Yamamoto M., Tseng A., Ajiki T., Saitoh Y. (1995). Extended cholecystectomy for carcinoma of the gallbladder. World J. Surg..

[9] Higuchi R., Yazawa T., Uemura S., Matsunaga Y., Ota T., Araida T., Furukawa T., Yamamoto M. (2020). Examination of Prognostic Factors Affecting Long-Term Survival of Patients with Stage 3/4 Gallbladder Cancer without Distant Metastasis. Cancers.

[10] Valle J., Wasan H., Palmer D.H., Cunningham D., Anthoney A., Maraveyas A., Madhusudan S., Iveson T., Hughes S., Pereira S.P. (2010). Cisplatin plus gemcitabine versus gemcitabine for biliary tract cancer. N. Engl. J. Med..

[11] Markussen A., Jensen L.H., Diness L.V., Larsen F.O. (2020). Treatment of Patients with Advanced Biliary Tract Cancer with Either Oxaliplatin, Gemcitabine, and Capecitabine or Cisplatin and Gemcitabine-A Randomized Phase II Trial. Cancers.

[12] Lamarca A., Palmer D.H., Wasan H.S., Ross P.J., Ma Y.T., Arora A., Falk S., Gillmore R., Wadsley J., Patel K. (2019). ABC-06|A randomised phase III, multi-centre, open-label study of active symptom control (ASC) alone or ASC with oxaliplatin/5-FU chemotherapy (ASC + mFOLFOX) for patients (pts) with locally advanced/metastatic biliary tract cancers (ABC) previously-treated with cisplatin/gemcitabine (CisGem) chemotherapy. J. Clin. Oncol..

[13] Pape U.F., Kasper S., Meiler J., Sinn M., Vogel A., Muller L., Burkhard O., Caca K., Heeg S., Buchner-Steudel P. (2020). Efficacy and Safety of CAP7.1 as Second-Line Treatment for Advanced Biliary Tract Cancers: Data from a Randomised Phase II Study. Cancers.

[14] Tella S.H., Kommalapati A., Borad M.J., Mahipal A. (2020). Second-line therapies in advanced biliary tract cancers. Lancet Oncol..

[15] Mahipal A., Tella S.H., Kommalapati A., Yu J., Kim R. (2020). Prevention and treatment of FGFR inhibitor-associated toxicities. Crit. Rev. Oncol. Hematol..

[16] Fostea R.M., Fontana E., Torga G., Arkenau H.T. (2020). Recent Progress in the Systemic Treatment of Advanced/Metastatic Cholangiocarcinoma. Cancers.

[17] Chakrabarti S., Kamgar M., Mahipal A. (2020). Targeted Therapies in Advanced Biliary Tract Cancer: An Evolving Paradigm. Cancers.

[18] Wijetunga I., McVeigh L.E., Charalambous A., Antanaviciute A., Carr I.M., Nair A., Prasad K.R., Ingram N., Coletta P.L. (2020). Translating Biomarkers of Cholangiocarcinoma for Theranosis: A Systematic Review. Cancers.

[19] Hogdall D., Lewinska M., Andersen J.B. (2018). Desmoplastic Tumor Microenvironment and Immunotherapy in Cholangiocarcinoma. Trends Cancer.

[20] Gentilini A., Pastore M., Marra F., Raggi C. (2018). The Role of Stroma in Cholangiocarcinoma: The Intriguing Interplay between Fibroblastic Component, Immune Cell Subsets and Tumor Epithelium. Int. J. Mol. Sci..

[21] Caligiuri A., Pastore M., Lori G., Raggi C., Di Maira G., Marra F., Gentilini A. (2020). Role of Chemokines in the Biology of Cholangiocarcinoma. Cancers.

[22] Malenica I., Donadon M., Lleo A. (2020). Molecular and Immunological Characterization of Biliary Tract Cancers: A Paradigm Shift Towards a Personalized Medicine. Cancers.

